# Advantages of the division of labour for the long-term population dynamics of cyanobacteria at different latitudes

**DOI:** 10.1098/rspb.2012.0755

**Published:** 2012-06-13

**Authors:** Valentina Rossetti, Homayoun C. Bagheri

**Affiliations:** Institute of Evolutionary Biology and Environmental Studies, University of Zurich, 8057 Zurich, Switzerland

**Keywords:** bacterial evolution, nitrogen fixation, photosynthesis, circadian rhythm, terminal differentiation

## Abstract

A fundamental advancement in the evolution of complexity is division of labour. This implies a partition of tasks among cells, either spatially through cellular differentiation, or temporally via a circadian rhythm. Cyanobacteria often employ either spatial differentiation or a circadian rhythm in order to separate the chemically incompatible processes of nitrogen fixation and photosynthesis. We present a theoretical framework to assess the advantages in terms of biomass production and population size for three species types: terminally differentiated (heterocystous), circadian, and an idealized species in which nitrogen and carbon fixation occur without biochemical constraints. On the basis of real solar irradiance data at different latitudes, we simulate population dynamics in isolation and in competition for light over a period of 40 years. Our results show that in isolation and regardless of latitude, the biomass of heterocystous cyanobacteria that optimally invest resources is comparable to that of the idealized unconstrained species. Hence, spatial division of labour overcomes biochemical constraints and enhances biomass production. In the circadian case, the strict temporal task separation modelled here hinders high biomass production in comparison with the heterocystous species. However, circadian species are found to be successful in competition for light whenever their resource investment prevents a waste of fixed nitrogen more effectively than do heterocystous species. In addition, we show the existence of a trade-off between population size and biomass accumulation, whereby each species can optimally invest resources to be proficient in biomass production or population growth, but not necessarily both. Finally, the model produces chaotic dynamics for population size, which is relevant to the study of cyanobacterial blooms.

## 1. Introduction

Division of labour generally helps an organism to efficiently integrate distinct cellular activities. Aquatic eukaryotes such as Volvocine algae exhibit a germ–soma division of labour, where somatic cells are used for motility, and germ cells are the non-motile reproductive units [1]. In Myxobacteria, cells of the fruiting body differentiate into spores, which resist starvation [2]. Cyanobacteria represent another relevant example of division of labour in prokaryotes. Cyanobacteria are among the most ancient phyla of photoautotrophic bacteria. They make use of solar light for oxygenic photosynthesis, and many species (diazotrophs) are able to fix nitrogen. A large part of global nitrogen fixation is owing to diazotrophic species [3–5], and cyanobacteria are also important oxygen producers [6]. Interestingly, oxygen produced by photosynthesis inhibits the nitrogenase enzyme required for nitrogen fixation. Cyanobacteria evolved different strategies to overcome this chemical incompatibility. Terminally differentiated cyanobacteria employ a division of labour in space, whereby nitrogen fixation is confined to somatic cells (heterocysts) endowed with a thick cell wall. Nitrogen fixation can be found also in non-heterosystous cyanobacteria [7]. Some species simply adapt to the immediate environment, with a temporal division of labour that follows the external day/night cycle. Moreover, some other species alternate photosynthesis and nitrogen fixation according to an endogenous clock, referred to as circadian rhythm [8,9]. Additionally, anoxigenic photosynthesis in sulphidic anaerobic environments has been reported in some species of the genus *Oscillatoria* [10,11]. A separate case is represented by *Trichodesmium* colonies, in which clusters of diazotrophic cells at the centre of the colonies provide a micro-oxic environment suitable for nitrogen fixation [12,13]. Nitrogen and carbon are dynamically stored in specialized granules [14], or as glycogen, respectively. Size and number of these structures can be affected by the external light/dark cycle and by the timing of nitrogen fixation [15,16]. Moreover, the ability of cyanobacteria to optimize polysaccharide formation and to adapt to changing daylength can represent an advantage over algal species in eutrophic aquatic environments [14]. Storage granules are also taken into account in several explanations proposed to influence cyanobacterial success [17].

The versatility of cyanobacteria allows them to be ubiquitous on our planet, although the availability of solar light varies markedly according to latitude and season. They have been detected in very different ecosystems such as marine and freshwater basins [4,18–21], polar and sub-polar regions [22,23], permafrost [24] and hot springs [25,26].

The ecology and distribution of cyanobacterial species have been studied experimentally in many distinct habitats [27–29]. However, theoretical works providing general explanations for their global distribution are more rare. Models are usually very detailed and focus on a single case study or a distinct habitat. For example, marine ecosystem models for emergent phytoplankton and nitrogen fixers' community structure involve complex parametrization and sophisticated equation systems [5,30]. Additional examples are competition between nitrogen-fixing and non-nitrogen-fixing species for light and nitrogen [31], comparison of circadian species with different free-running periods [32], or population dynamics and nitrogen fixation rates in the Baltic Sea [22,33–35].

Terminally differentiated cyanobacteria represent an important example of a primitive form of division of labour between germline and somatic cells. This kind of differentiation was first observed and studied in the eukaryotic order Volvocales [36], and could evolve along several evolutionary paths [37] as a consequence of a trade-off between reproduction and survival [38]. More recent modelling approaches applicable to eukaryotes and prokaryotes investigated the most advantageous proportion of somatic cells in multi-cellular organisms [39] and the possibility of rapid evolution of division of labour as a consequence of developmental plasticity [40].

This paper extends the work developed in Rossetti *et al.* [41]. We investigated the performance of undifferentiated and differentiated cyanobacteria in terms of population size and biomass production, when they grow in isolation as well as when they compete for light. This can elucidate some of the advantages that each strategy for division of labour provides for the resolution of the biochemical incompatibility between nitrogen and carbon fixation. Our theoretical work takes into account light availability at distinct geographical locations on the basis of real data of the solar irradiance on the Earth's surface. In addition, we model different consumption rates of the resources (fixed carbon and nitrogen) that are invested by the cells in reproduction and nitrogen fixation. The model of the two extant species types is then compared with the hypothetical scenario of a bacterial organism that is free from biochemical constraints on nitrogen fixation. Such comparisons can indicate to what extent different forms of division of labour help to overcome biochemical constraints.

## 2. Material and methods

### (a) Mathematical model

**Figure 1.**
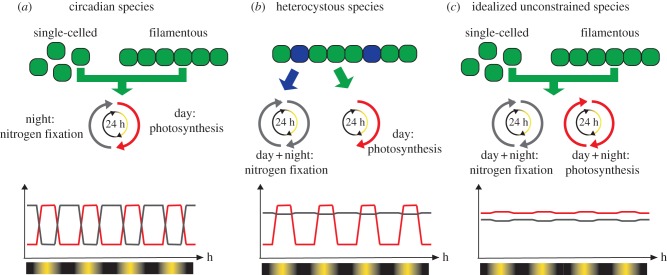
A schematic of the model species. (*a*) Undifferentiated cyanobacteria (both single-celled and filamentous species) fix carbon and nitrogen according to a circadian rhythm. All cells perform photosynthesis during the day and fix nitrogen during the night; hence, the two processes alternate on a day/night rhythm. (*b*) In terminally differentiated cyanobacteria, vegetative cells perform photosynthesis during the daytime (i.e. when light is available). Heterocyst cells provide an anoxic environment where nitrogen fixation can take place at any time, and hence is limited only by the availability of resources. (*c*) In the idealized species, nitrogen and carbon fixation can be performed without temporal inhibitory restrictions and are limited only by the availability of substrates. Oscillation amplitude and position of the red and grey lines for carbon and nitrogen fixation are arbitrary and for illustrative purposes only. Green circles, vegetative cell; blue circles, heterocystous cell; red line, carbon fixation; grey line, nitrogen fixation; black line, night; yellow line, day.

We developed a model of cyanobacterial populations that survive through the production and exchange of fixed carbon and nitrogen. We model two cyanobacterial species types and an idealized species used as a hypothetical case, as shown in figure 1. The cyanobacterial species types correspond to undifferentiated (circadian) and terminally differentiated (heterocystous) species. In the former, carbon and nitrogen fixation alternate according to a circadian rhythm. In the latter, they are spatially separated. Although we do not explicitly model a circadian clock at the genetic level, our use of the term circadian refers to species that possesses a true internal circadian rhythm. The species in the idealized model has no biochemical constraints, whereby in each cell, nitrogen and carbon fixation can be performed whenever substrates are available. The performance of each species type is measured in terms of biomass production and population size. The dependence on geographical location is expressed through the latitude and is included in a function for solar irradiance based on real data of irradiance on the Earth's surface. The resource investment of the cell in reproduction and nitrogen fixation occurs at variable rates, represented by parameters *r* and *a*, respectively. All models consist of a set of ordinary differential equations that were run for a number of iterations corresponding to 40 years. Each species was evaluated at five latitudes ranging from 0 to 60 N, for 13 values of parameter *r* ranging from 10^−5^ to 10 fmol cells^−1^ h^−1^ and for five values of parameter *a* ranging from 0.05 to 5 fmol cells^−1^ h^−1^. Assuming a symmetry of environmental conditions between Northern and Southern Hemispheres, the model takes into account only northern latitudes. In the following, we present the systems of equations representing each species type. Concentrations of vegetative and heterocystous cells are indicated with *V* and *H*, respectively. Concentrations of nitrogen and carbon are represented by *N* and *C*, respectively. Default values and units of model parameters are listed in table 1.

**Table 1.** Parameters values used in the simulations. (For parameters *r* and *a* ranges are indicated. E, Einstein; PSU, photosynthetic units.)

parameter description	symbol	value	unit
investment in reproduction	*r*	10^−5^–10	fmol cell^−1^ h^−1^
investment in N-fixation	*a*	0.05–5	fmol cell^−1^ h^−1^
decay rate	*p*_3_	0.001	h^−1^
maximum irradiance	*A*	1000	µΕ m^−2^ h^−1^
half saturation constant	k	100	fmol µm^−3^
total stoich. concentration	*r*_0_	1	—
first-order rate constant	*k*_0_	1	—
N specificity constant	*k*_N_	10	fmol^−1^ µm^3^ h^−1^
C specificity constant	*k*_C_	10	fmol^−1^ µm^3^ h^−1^
N-C specificity constant	*k*_NC_	1	(fmol^−1^ µm^3^)^2^ h^−1^
irradiance specificity constant	*k*_I_	10	µΕ^−1^m^2^
PSU specificity constant	*k*_G_, *k*_V_	10	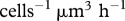
irradiance-PSU specificity constant	*k*_IG_, *k*_IV_	1	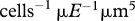
photosynthesis rate	*r*_p_	6	fmol µm^−3^ h^−1^
cell reproduction rate	*r*_d_	0.03	h^−1^

### (b) Circadian species

This model represents undifferentiated cyanobacteria with a circadian programme. It does not distinguish between single-celled and filamentous species, as it considers both species as a set of circadian-regulated cells producing and sharing carbon and nitrogen. Nitrogen fixation and photosynthesis are regulated by an internal clock that follows daily light availability. The endogenous rhythm of the cellular activities is expressed by the functions 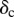 and 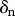: these functions are multiplied by the carbon and nitrogen fixation terms and inhibit these processes during night and day, respectively. The system of equations is given by
2.1
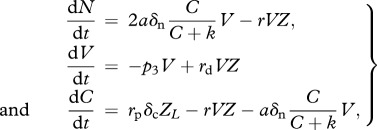

where
2.2
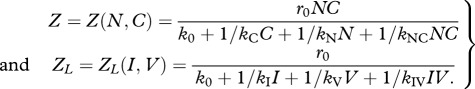



### (c) Heterocystous species

In this model, we consider terminally differentiated species. These possess heterocystous cells, where nitrogenase is protected from oxygen. Hence, the nitrogen production term does not have any inhibition coefficient 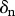; however, only heterocysts can fix nitrogen. Carbon fixation follows the external light/dark alternation. Vegetative cells differentiate into heterocysts with a rate *p*_h_ = 1 − *p*_v_. The default value *p*_v_ = 0.85 was chosen as an approximation of the values observed in nature. The set of equations is given by
2.3
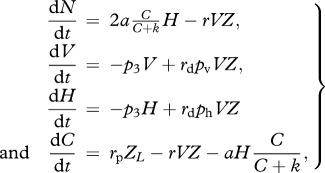

where *Z* and *Z*_*L*_ are as in equation (2.2).

### (d) Idealized unconstrained species

This hypothetical species type represents an ideal cyanobacterial species in which biochemical constraints owing to the inhibition of oxygen on nitrogenase are absent. This implies that nitrogen and carbon fixations can take place in each cell and at any time, as soon as the necessary resources are available. No such species is known. The system of equations describing this model is similar to that of the circadian species; however, nitrogen and carbon fixations are not regulated by the 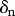 and 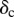 functions anymore:
2.4
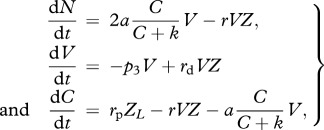

where *Z* and *Z*_*L*_ are as in equation (2.2).

### (e) Model explanation

In systems (2.1), (2.3) and (2.4), the parameter *r* is the rate at which nitrogen and carbon are invested per cell division. Carbon is consumed at rate *a* to be invested in nitrogen fixation. Nitrogen is fixed at a rate 2*a*. The reproduction term is represented by the function *Z*, whose substrates are carbon and nitrogen. Carbon fixation is expressed by the function *Z*_*L*_, which depends on the irradiance *I* and on the vegetative cells (considered as photosynthetic units). Because solar light depends only on time, but is not an explicit variable of the system, the use of photosynthetic units as a limiting factor prevents the exponential growth of the population. The reader can refer to Rossetti *et al.* [41] for a more detailed description of the equation structure.

Solar light is represented by *I*, and an antilogistic function [42] is given by
2.5
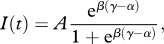

where
2.6
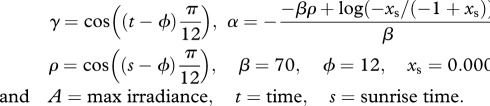



The value of maximum irradiance *A* depends on the month and on the latitude and it is based on real data (courtesy of the Swiss Federal Research Institute WSL, Birmensdorf). The sunrise time is determined according to day and latitude (timeanddate.com/worldclock/sunrise.html). Plots of the irradiance function and details regarding the approximation of real data with the function *I* can be found in the electronic supplementary material.

Circadian species are characterized by an endogenous clock that regulates the timing of carbon and nitrogen fixation. Similar to the irradiance case, we use antilogistic functions to model carbon and nitrogen fixation rhythm, expressed by 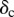 and 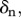 respectively:
2.7
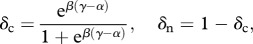

where **γ**, **α** and **ρ** are as in equation (2.6).

### (f) Performance measures

All differential equations were numerically integrated with Matlab. Unless stated otherwise, each species was simulated separately in order to determine its performance without inter-species competition. The performance of the populations was measured in two ways: first as total population size (vegetative cells for circadian and unconstrained species, vegetative plus heterocysts for the differentiated species); second, as biomass accumulation. The proxy for biomass in this case is the total amount of carbon given by
2.8
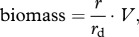

where *V* is the population size expressed as number of cells per unit volume. In equation (2.8), the ratio 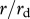 between the cell investment of carbon for reproduction and the cell division rate represents the amount of carbon produced per cell. By multiplying this ratio for the total number of cells, we obtain the total amount of carbon (measured in fmol 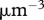).

## 3. Results

### (a) Population dynamics

**Figure 2.**
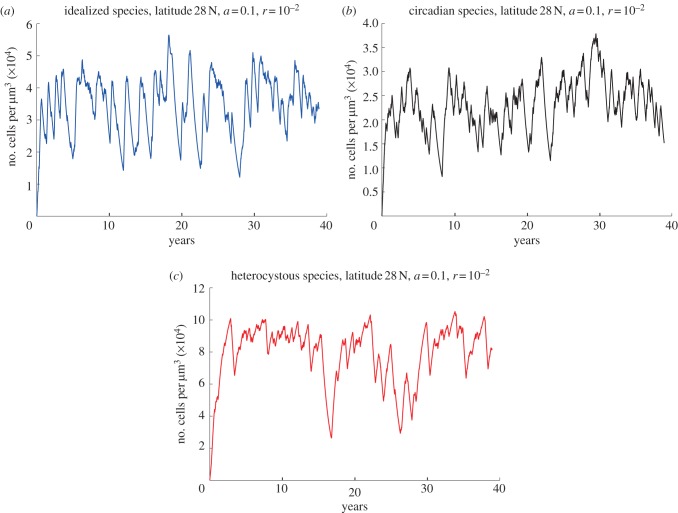
Sample runs of the three modelled species for a number of iterations corresponding to 40 years.

The model developed for this work is entirely deterministic, though the analytical complexity of the equations allows only for a numerical study of their dynamics. Figure 2 shows the daily population size over 40 years for sample single runs. The system does not reach a steady state, rather it presents a dynamic characterized by oscillations and peaks similar to that of chaotic systems. This can be explained by the fact that although the pattern of light availability is repeated identically every year, the population size at the beginning of each yearly cycle differs from the one of the previous year. Moreover, the speed at which light availability changes is higher than the speed at which the cyanobacteria population can equilibrate at the new external conditions. Hence, a steady state cannot be reached.

### (b) Effect of latitude and cell investment on performance

**Figure 3.**
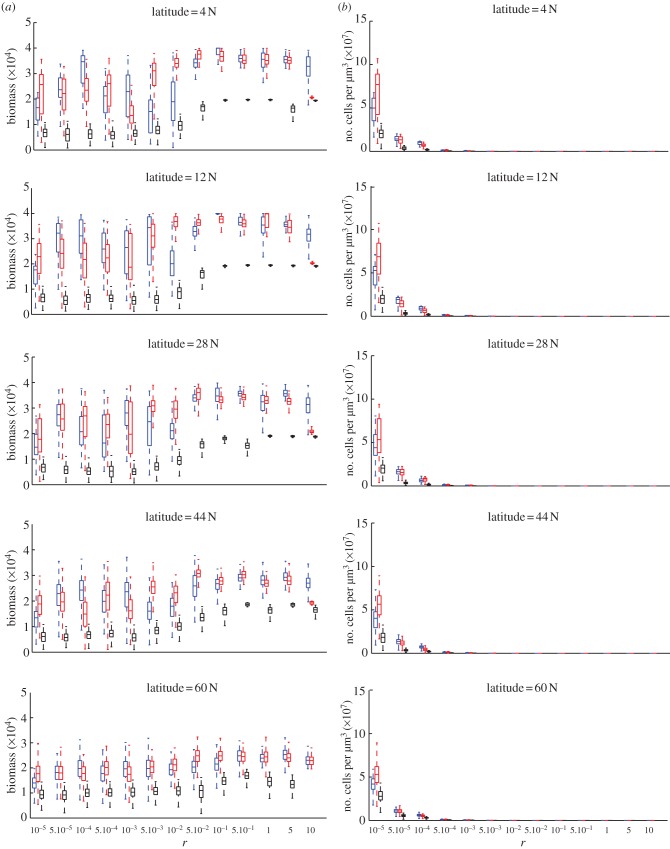
Box plot of (*a*) the biomass production and (*b*) population size computed daily over the last 35 years of a simulated 40 year period at different latitudes and values of cell investment *r*. Biomass is expressed in fmol µm^−3^. In each box, the central mark is the median, the horizontal edges of the box are the 25th and 75th percentiles and the whiskers extending to the most extreme data points are not considered outliers (outliers not shown). Blue line, unconstrained; red line, heterocystous; black line, circadian.

In figure 3, box plots of biomass and number of cells over a 35 years period are shown at different values of *r* and latitude. Of the total 40 years shown in figure 2, we discarded the first 5 years in order to exclude the initial exponential growth phase and have a more homogeneous dataset for the box plots. The parameter *r* can be interpreted as the rate of nitrogen and carbon investment into population growth per cell division. For each value of *r* and latitude, data are plotted for the value of *a* that provides the highest mean biomass. The parameter *a* can be interpreted as the rate of carbon investment in nitrogen fixation.

Up to 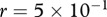, the population biomass of all three species generally increases with increasing *r*. For 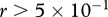, biomass flattens or even decreases. This trend is less marked at latitude 60 N. While the biomass of heterocystous and unconstrained model species ranges between comparable values, that of circadian species is clearly lower than the others.

The trend of the number of cells is very different from that of biomass. For all species, the number of cells decreases rapidly with increasing values of *r*. Circadian species always exhibit lower values than the other two species types. For the smallest value of *r*, heterocystous species reach a higher population size than do the unconstrained species.

### (c) Performance comparison at optimal cell investment

**Figure 4.**
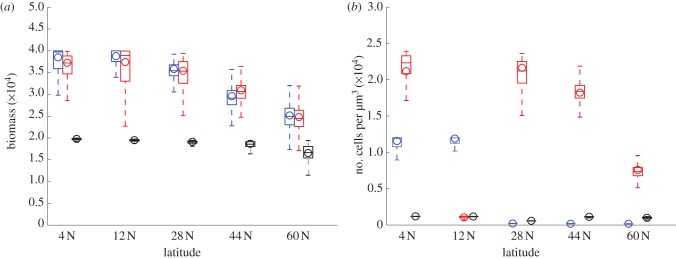
(*a*) A comparison of the box plots corresponding to the (*a, r*) providing the highest biomass at each latitude for each species. Parameter *r* represents the resource investment into reproduction, *a* is the resource investment in nitrogen-fixation. Both *r* and *a* are measured in fmol cell^−1^ h^−1^ and biomass is expressed in fmol µm^−3^. Data correspond to daily biomass over a 35 year period. (*b*) Box plot of the number of cells over the last 35 years of a simulated 40 year period obtained with the same (*a, r*) values as in panel (*a*). The open circle in each box plot corresponds to the mean values of biomass and number of cells, respectively. Blue, unconstrained; red, heterocystous and black, circadian.

For each latitude and species type, there is a pair of *a* and *r* values that provide a maximum average biomass. We refer to these values as optimal (*a, r*) pairs. In figure 4*a*, we compare the biomass distribution of the two species obtained with the optimal pairs at each latitude. Open circles indicate the mean biomass values used to determine the corresponding optimal pair. Figure 4*b* shows the population size of the two species obtained at each latitude with the same optimal (*a, r*) pairs determined for the biomass. At optimal (*a, r*) values, heterocystous and unconstrained species reach comparable levels of biomass production at every latitude. The biomass of circadian species is at lower levels in all cases. For the same (*a*, *r*) pairs however, the heterocystous species reach the highest number of cells, except for latitude 12 N. At latitudes 4 N and 12 N, the number of cells of the unconstrained species exceeds that of circadian species.

### (d) Best cell investment for biomass and population size

**Figure 5.**
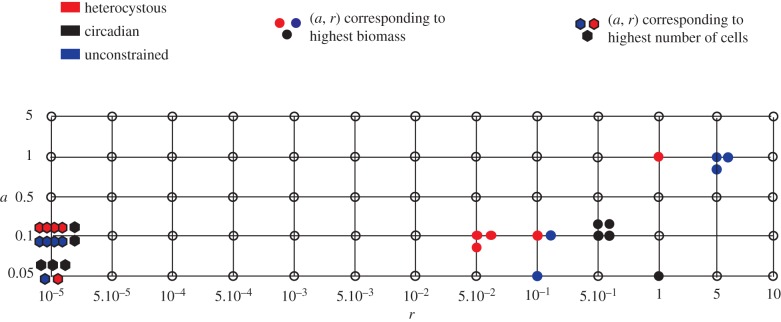
Best cell investment for each species and latitude. Coloured dots and diamonds indicate that the corresponding grid point is an optimal (*a, r*) pair that provides the highest biomass and population size, respectively, at a given latitude (table 2).

Figure 5 shows a grid of the five values of *a* and the 13 values of *r* used in the simulations. The presence of a filled dot on a grid point indicates that a species (type indicated by the colour code) at a given latitude reaches its best performance in terms of biomass at the corresponding (*a, r*) pair. The (*a, r*) pair values and the corresponding latitudes are listed in table 2. The biomass of circadian and heterocystous species is optimal for low values of *a* and high values of *r*. For the unconstrained species, the range of optimal *a* and *r* values is slightly bigger than that of the other two species. On the whole, each species has the highest biomass production for at most three grid points.

**Table 2:** Best (*a*,*r*) pair that provides the highest average biomass at each latitude.

	circadian	unconstrained	heterocystous
latitude 4 N	(0.1, 5 × 10^−1^)	(0.05, 10^−1^)	(0.1, 5 × 10^−2^)
latitude 12 N	(0.1, 5 × 10^−1^)	(0.1, 10^−1^)	(1, 1)
latitude 28 N	(0.05, 1)	(1,5)	(0.1, 5 × 10^−2^)
latitude 44 N	(0.1, 5 × 10^−1^)	(1, 5)	(0.1, 5 × 10^−2^)
latitude 60 N	(0.1, 5 × 10^−1^)	(1, 5)	(0.1, 10^−1^)

The presence of a filled diamond on a grid point indicates that a species at a given latitude reaches its best performance in terms of population size at the corresponding (*a, r*) pair. It can be observed that all three species reach their highest number of cells at only two grid points.

### (e) A trade-off between biomass and population size

From figure 3, it can be seen that for all species populations, biomass production increases with increasing *r*, while the number of cells decreases with increasing *r*. In figure 4, species that have comparable biomass production for their optimal (*a, r*) pair can have very different population sizes. Figure 5 summarizes these findings, showing that the (*a, r*) pairs corresponding to the highest biomass always differ from those corresponding to the highest population size. We can hence infer the existence of a trade-off between biomass production and population growth in terms of number of cells. To mathematically verify this hypothesis, we computed the steady-state population size V* for system (2.1) and any given *r*, denoted as V* (*r*). We allow *r* to vary and fix values of irradiance, the functions 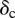 and 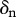, and all other parameters (listed in the electronic supplementary material). The same approach would be also valid for systems (2.3) and (2.4). We then computed the sensitivity coefficient 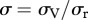, where 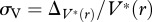 and 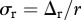. The value of **σ** is an indicator of response of population size to the variation of *r*. For the range of *r* values considered in this model, we found that 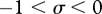, and that **σ** decreases towards −1 with increasing *r*. When 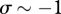, the absolute value of the proportional change in V* equals that of the proportional change in *r*; hence, no trade-off is expected. When the value of **σ** is far from −1, the response of the population size V* to changes in *r* is weaker, indicating the existence of a trade-off. This implies that the decrease of biomass per cell owing to a smaller *r* cannot be compensated linearly by an increase in the population size. Details are provided in the electronic supplementary material.

### (f) Inter-species competition

We simulated the growth of circadian and heterocystous species types when they compete for solar light. The corresponding set of differential equations is in the electronic supplementary material, section S3. In this case, each species type produces and consumes its own resources, while the available solar energy is shared among the two species types. Pairwise competitions were run for a wide range of (*a, r*) values at every latitude (data not shown). We found that when the value of *a* for the circadian type is lower that the value of *a* for the heterocystous type, circadian species can win the competition. Five examples of such cases are shown in the electronic supplementary material, figure S4.

## 4. Discussion

### (a) Long-term population dynamics

The sample runs in figure 2 show that the long-term population dynamics superficially resemble a stochastic system, although no stochastic term is present in our models. Each species continuously tries to catch up with the external light availability and has a different population size at the beginning of each new seasonal cycle. The irregular oscillations and the isolated peaks of the population size are suggesting chaotic dynamics and could be part of the explanation for the difficult predictability of cyanobacterial blooms observed in many bodies of water.

### (b) Cell differentiation and nitrogenase sensitivity to oxygen

The unconstrained model represents a hypothetical, ideal combination of nitrogen and carbon fixation. In this hypothetical species, the nitrogenase enzyme is not sensitive to oxygen, allowing for an unconstrained coexistence of the two processes. Conversely, the circadian model simulated in this work is subject to very strict constraints. The inhibitory functions 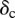 and 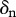 stop carbon and nitrogen fixation during night and day, respectively. Heterocystous species solve this biochemical incompatibility problem by developing oxygen-protected cells in which nitrogenase can be constantly expressed. The results shown in figures 3 and 4 indicate that heterocystous and unconstrained species have comparable performance in terms of biomass production. For every value of *r* in figure 3*a* and for every optimal (*a, r*) pair in figure 4*a*, the biomass value range of the two species overlap. The biomass of circadian species is instead considerably lower, although the value ranges partially overlap with that of the other species at latitude 60 N and for small values of *r* at lower latitudes (figure 3*a*). These outcomes indicate that cell differentiation is a successful way to circumvent the biochemical constraints, because it allows for a biomass production that is comparable with the ideal unconstrained species. In the absence of exogenous sources of fixed nitrogen, spatial division of labour can hence significantly improve biomass accumulation. The scarce performance of circadian species can be explained by the fact that in this model, the endogenous rhythm and associated inhibition cycle are very strict and does not allow for coexistence between carbon and nitrogen fixation.

In many cyanobacteria, division of labour is missing, and nitrogen fixation cannot be performed. One interesting future direction would be the study of the advantages of non-nitrogen-fixers over nitrogen-fixing species. An attempt has been made by Agawin *et al.* [31] Their model and experiments study the role of different physiological processes in the determination of the abundance of nitrogen fixers and non-nitrogen-fixers in oligotrophic ocean water.

### (c) Trade-off between biomass production and population growth

As shown in figures 3 and 4 and summarized in figure 5, there is a trade-off between biomass production and population size whereby given a value of carbon and nitrogen consumption, cyanobacteria can have either a high biomass output or a large population size, but not both. Interestingly, the values of *r* at which the trade-off is higher (i.e. 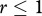, see the electronic supplementary material, figure S3) correspond to the domain in which increasing *r* will lead to higher biomass production. This explains why in figure 5, the optimal biomass production of heterocystous and circadian species is not at the largest *r*-value (*r* = 10), but rather in the range 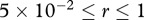. Above a certain value of *r*, the increase in the cell investment does not lead to an increase in biomass anymore. The reasons for the presence of the trade-off can be found in the form of the function *Z* = *Z*(*N, C*) regulating resource uptake and cell division. Because *Z* is a saturation function of carbon and nitrogen, excess nutrients can be incorporated into cell division only up to a certain level, above which population growth (cell division) cannot be increased by decreasing the investment per cell.

### (d) Ocean mixing and species maintenance

While growth in isolation favours high performance of heterocystous species in terms of population size and biomass, inter-species competition for solar light indicates some conditions under which circadian species reach a higher population size and biomass. Circadian species can succeed in competitions whenever their investment of carbon and the corresponding nitrogen fixation activity are such that a waste of nitrogen is prevented and growth is assured (see the electronic supplementary material, section S3 and figure S4).

Oceans, and more in general water bodies, are subjected to water mixing. This could favour the migration of species types to latitudes that differ from those where they successfully thrive, hence contributing to the maintenance of the species diversity observed in nature.

## 5. Conclusions

This work shows that in cyanobacteria, spatial division of labour can overcome the biochemical constraints owing to nitrogenase oxygen sensitivity and can enhance biomass production. In terms of success at biomass accumulation, heterocystous species grown in isolation can become comparable to an idealized species that has no biochemical constraints. A strict temporal division of labour, on the other hand, leads to a lower biomass productivity. This suggests that in circadian species that lack outside sources of fixed nitrogen, selection would have to act to reduce the sensitivity of nitrogenase enzyme to oxygen in order to increase biomass productivity. This would allow for a more flexible temporal regulation of carbon and nitrogen fixation. However, we also show that owing to a trade-off between population size and biomass, different species can be successful in terms of different performance measures. The competition simulations also showed how an allocation of resources that prevents a waste of fixed nitrogen is determining the winning species in competition. Heterocsytous species that invest more carbon into nitrogen fixation might be fast-growing in an initial phase, but on the long term, can be outcompeted by circadian species that invest less. These arguments could potentially be involved in explanations for the coexistence of different species types in the same environment and for the species variety all over the globe. Finally, the oscillating dynamics of the population size could open new perspectives in the study of cyanobacterial blooms.
